# Supplementary material for: Chromosome segregation synchrony in S. pombe is noise limited and arises without positive feedback
Source: J Cell Biol. 2026 May 14;225(7):e202602088. doi: 10.1083/jcb.202602088 (PMC13175033; doi:10.1083/jcb.202602088)
Supplement: Table S1 — shows statistical analysis of experimental results. [file jcb_202602088_tables1.docx]

Table S1 - Statistical analysis of experimental results

|  |  | **n** | **mean (sec)** | **median (sec)** | **stdev (sec)** | **p-value (Kolmogorov-Smirnov)** | **tested against** |
| --- | --- | --- | --- | --- | --- | --- | --- |
| **WT (rich medium)** | chromosome I vs. II | 132 | -6.77 | -8 | 16.41 |  |  |
|  | chromosome III vs. II | 86 | 11.5 | 13 | 16.89 |  |  |
| **WT (minimal medium)** | chromosome I vs. II | 126 | -3.44 | -1.01 | 19 | 0.1641 | chromosome I vs. II WT (rich medium) |
|  | chromosome III vs. II | 145 | 11.47 | 12.99 | 13.51 | 10.3891  21.56e-14 | 1chromosome III vs. II WT (rich medium)  2chromosome I vs. II WT (minimal medium) |
| **imr1L / dh1R** | imr1L vs. dh1R | 78 | 0.2 | 0 | 7.3 |  |  |
|  | imr1L vs. dh1R | 88 | 0.9 | 0 | 4.1 |  |  |
| **WT central centromere tags** | chromosome I vs. II | 137 | -3.49 | -3.5 | 17.41 | 0.4819 | chromosome I vs. II WT (minimal medium) |
|  | chromosome III vs. II | 146 | 7.77 | 7.12 | 18.87 | 0.0291 | chromosome III vs. II WT (minimal medium) |
| **separase mutant**  **(*cut1-206,* rich medium)** | chromosome I vs. II | 87 | -7.46 | -8 | 40.55 | 0.0484 | chromosome I vs. II WT (rich medium) |
|  | chromosome III vs. II | 71 | 32.69 | 34 | 31.89 | 7.65e-09 | chromosome III vs. II WT (rich medium) |
| **separase mutant (*cut1-206,* rich to minimal medium)** | chromosome I vs. II | 74 | -4.4 | -3.5 | 28.52 | 10.0361  20.7575 | 1chromosome I vs. II WT (rich medium)  2chromosome I vs. II separase  mutant (rich medium) |
|  | chromosome III vs. II | 188 | 24.61 | 24.5 | 34.51 | 12.45e-9  20.1219 | 1chromosome III vs. II WT (rich medium)  2chromosome III vs. II separase mutant (rich medium) |
| **separase mutant (*cut1-206,* combined results)** | chromosome I vs. II | 161 | -6.05 | -8 | 35.46 | 10.0185  20.9993  30.9962 | 1chromosome I vs. II WT (rich medium)  2chromosome I vs. II separase mutant (rich medium) 3chromosome I vs. II separase mutant (rich to minimal medium) |
|  | chromosome III vs. II | 259 | 26.83 | 28 | 33.94 | 12.94e-11  20.4016  30.9798 | 1chromosome III vs. II WT (rich medium)  2chromosome III vs. II separase mutant (rich medium) 3chromosome III vs. II separase mutant (rich to minimal medium) |
| **securin overexpression** | chromosome I vs. II | 62 | -11.29 | -14 | 21.58 | 0.0021 | chromosome I vs. II WT (minimal medium) |
|  | chromosome III vs. II | 95 | 9.95 | 10.5 | 13 | 0.3788 | chromosome III vs. II WT (minimal medium) |
| **securin/separase overexpression** | chromosome I vs. II | 99 | -8.63 | -7 | 19.56 | 10.0130  20.0239 | 1chromosome I vs. II securin overexpression 2chromosome I vs. II WT (minimal medium) |
|  | chromosome III vs. II | 60 | 7.53 | 3.5 | 18.81 | 10.1242  20.0020 | 1chromosome III vs. II securin overexpression 2chromosome III vs. II WT (minimal medium) |

| **WT + 3 ug/mL MBC (minimal medium)** | chromosome I vs. II | 67 | -11.54 | -7 | 21.78 | 0.0007 | chromosome I vs. II WT (minimal medium) |
| --- | --- | --- | --- | --- | --- | --- | --- |
|  | chromosome III vs. II | 62 | 14.79 | 10.5 | 22.1 | 0.0896 | chromosome III vs. II WT (minimal medium) |
| **klp5Δ** | chromosome I vs. II | 56 | -3.69 | -1.75 | 18.63 | 0.8037 | chromosome I vs. II WT (minimal medium) |
|  | chromosome III vs. II | 37 | 13.15 | 10.5 | 14.3 | 0.3322 | chromosome III vs. II WT (minimal medium) |
| **ΔN-cyclin B nuclear division**  **(minimal medium)** | chromosome I vs. II | 101 | -5.1 | -7 | 18.12 | 0.2635 | chromosome I vs. II WT (minimal medium) |
| **ΔN-cyclin B no nuclear division**  **(minimal medium)** | chromosome I vs. II | 98 | -5.95 | -8 | 24.6 | 10.2377  20.8248 | 1chromosome I vs. II WT (minimal medium)  2ΔN-cyclin B nuclear division |
| **APC/C mutant**  **(*cut9-665*, rich medium)** | chromosome I vs. II | 158 | -11.9 | -12.25 | 43.51 | 0.0046 | chromosome I vs. II WT (rich medium) |
|  | chromosome III vs. II | 161 | 18.79 | 17.5 | 24.99 | 0.0002 | chromosome III vs. II WT (rich medium) |
| **proteasome inhibition (minimal medium)** | chromosome I vs. II | 131 | -9.62 | -14 | 91.8 | 7.58e-07 | chromosome I vs. II WT (minimal medium) |
|  | chromosome III vs. II | 153 | 41.07 | 35 | 52.67 | 4.24e-20 | chromosome III vs. II WT (minimal medium) |
| **marker swap (cen1-lacO**  **/ cen2-tetO)** | chromosome I vs. II | 152 | -8.96 | -7 | 19.16 | 1.54e-04 | chromosome I vs. II WT (minimal medium) |
| **arm markers** | chromosome II, cen2 vs. arm | 79 | 120.75 | 119 | 46.07 | 1<2.23e-308  2<2.23e-308  30.1141 | 1chromosome I vs. II WT (minimal medium) 2chromosome III vs. II WT (minimal medium)  3central centromere II vs. arm |
|  | chromosome II, cnt2 vs. arm | 72 | 105.17 | 103.75 | 46.63 | 1<2.23e-308  2<2.23e-308 | 1chromosome I vs. II WT (minimal medium) 2chromosome III vs. II WT (minimal medium) |
