# Supplementary material for: Chromosome segregation synchrony in S. pombe is noise limited and arises without positive feedback
Source: J Cell Biol. 2026 May 14;225(7):e202602088. doi: 10.1083/jcb.202602088 (PMC13175033; doi:10.1083/jcb.202602088)
Supplement: Table S2 — shows S. pombe strains. [file jcb_202602088_tables2.docx]

**Table S2 - *S. pombe* strains**

Figure 1C-D

| SL239' | *h+* | *leu1- ade6-M216 cen2::lacO-kanR:ura+ his7+::P.dis1-GFP-lacI-NLS cen3::Scer\LEU2:tetO*  *zfs1+::natR:P.adh31-tetR-tdTomato* |
| --- | --- | --- |
| **Figure 1E** |  |  |
| SU495/SU496 | *h-* | *leu1- ura4-D18? FY397::RS imr1L::lacO-RS his7+::P.dis1-GFP-lacI-NLS dh1R::tetO:ura4+ zfs1+::natR:P.adh31-tetR-tdTomato* |
| **Figure 1F** |  |  |
| SI541 | *h+* | *leu1- ade6-M216 ura4-D18? cen2::lacO-kanR:ura+ his7+::P.dis1-GFP-lacI-NLS dh1L::ura4+:tetO zfs1+::natR:P.adh31-tetR-tdTomato* |
| SL239' | *h+* | *leu1- ade6-M216 cen2::lacO-kanR:ura+ his7+::P.dis1-GFP-lacI-NLS cen3::Scer\LEU2:tetO zfs1+::natR:P.adh31-tetR-tdTomato* |
| **Figure 1H** |  |  |
| SI541 | *h+* | *leu1- ade6-M216 ura4-D18? cen2::lacO-kanR:ura+ his7+::P.dis1-GFP-lacI-NLS dh1L::ura4+:tetO zfs1+::natR:P.adh31-tetR-tdTomato* |
| SL239' | *h+* | *leu1- ade6-M216 cen2::lacO-kanR:ura+ his7+::P.dis1-GFP-lacI-NLS cen3::Scer\LEU2:tetO zfs1+::natR:P.adh31-tetR-tdTomato* |
| PQ768/PQ768' | *h+* | *leu1- ura4-D18 cnt1::lacO his7+::P.dis1-GFP-lacI-NLS cnt2::tetO:ura4+ zfs1+::natR:P.adh31-tetR-tdTomato* |
| ST968/ST968' | *h+* | *leu1- ura4-D18 cnt3::Scer\LEU2:lacO his7+::P.dis1-GFP-lacI-NLS cnt2::tetO:ura4+ zfs1+::natR:P.adh31-tetR-tdTomato* |
| **Figure 2A-B** |  |  |
| SI541 | *h+* | *leu1- ade6-M216 ura4-D18? cen2::lacO-kanR:ura+ his7+::P.dis1-GFP-lacI-NLS dh1L::ura4+:tetO zfs1+::natR:P.adh31-tetR-tdTomato* |
| SL239' | *h+* | *leu1- ade6-M216 cen2::lacO-kanR:ura+ his7+::P.dis1-GFP-lacI-NLS cen3::Scer\LEU2:tetO zfs1+::natR:P.adh31-tetR-tdTomato* |
| SM388 | *h?* | *leu1- ade6-M21? ura4-D18? cen2::lacO-kanR:ura+ his7+::P.dis1-GFP-lacI-NLS dh1L::ura4+:tetO zfs1+::natR:P.adh31-tetR-tdTomato cut1-206* |
| SM387 | *h?* | *leu1- ade6-M21? cen2::lacO-kanR:ura+ his7+::P.dis1-GFP-lacI-NLS cen3::Scer\LEU2:tetO* |
|  |  | *zfs1+::natR:P.adh31-tetR-tdTomato cut1-206* |
| **Figure 2C-D** |  |  |
| SI541 | *h+* | *leu1- ade6-M216 ura4-D18? cen2::lacO-kanR:ura+ his7+::P.dis1-GFP-lacI-NLS dh1L::ura4+:tetO zfs1+::natR:P.adh31-tetR-tdTomato* |
| SL239' | *h+* | *leu1- ade6-M216 cen2::lacO-kanR:ura+ his7+::P.dis1-GFP-lacI-NLS cen3::Scer\LEU2:tetO zfs1+::natR:P.adh31-tetR-tdTomato* |
| SW562 | *h-* | *leu1+::P.cut1(long)-cut1+ cen2::lacO-kanR:ura+ his7+::P.dis1-GFP-lacI-NLS dh1L::ura4+:tetO zfs1+::natR:P.adh31-tetR-tdTomato natNT2:P.adh1(#6)-cut2-GFP(Y66L):kanR* |
| SW566 | *h-* | *leu1+::P.cut1(long)-cut1+ cen2::lacO-kanR:ura+ his7+::P.dis1-GFP-lacI-NLS cen3::Scer\LEU2:tetO zfs1+::natR:P.adh31-tetR-tdTomato natNT2:P.adh1(#6)-cut2-GFP(Y66L):kanR* |
| **Figure2E-F** |  |  |
| SI541 | *h+* | *leu1- ade6-M216 ura4-D18? cen2::lacO-kanR:ura+ his7+::P.dis1-GFP-lacI-NLS dh1L::ura4+:tetO zfs1+::natR:P.adh31-tetR-tdTomato* |
| SL239' | *h+* | *leu1- ade6-M216 cen2::lacO-kanR:ura+ his7+::P.dis1-GFP-lacI-NLS cen3::Scer\LEU2:tetO zfs1+::natR:P.adh31-tetR-tdTomato* |

| **Figure 3A-B** |  |  |
| --- | --- | --- |
| SL274 | *h+* | *leu1+::P.nmt81-cdc13Δ(1-67) ade6-M216 ura4-D18? cen2::lacO-kanR:ura+ his7+::P.dis1-GFP-lacI-NLS dh1L::ura4+:tetO zfs1+::natR:P.adh31-tetR-tdTomato* |
| SI541 | *h+* | *leu1- ade6-M216 ura4-D18? cen2::lacO-kanR:ura+ his7+::P.dis1-GFP-lacI-NLS dh1L::ura4+:tetO zfs1+::natR:P.adh31-tetR-tdTomato* |
| **Figure 3E-F** |  |  |
| SL249 | *h-* | *leu1- ade6-M216 dh1L::ura4+:tetO zfs1+::natR:P.adh31-tetR-tdTomato cut2-GFP:kanR* |
| SL275 | *h-* | *leu1+::P.nmt81-cut2Δ(1-75) ade6-M216 dh1L::ura4+:tetO zfs1+::natR:P.adh31-tetR-tdTomato cut2-GFP:kanR* |
| **Figure 4C** |  |  |
| SI541 | *h+* | *leu1- ade6-M216 ura4-D18? cen2::lacO-kanR:ura+ his7+::P.dis1-GFP-lacI-NLS dh1L::ura4+:tetO zfs1+::natR:P.adh31-tetR-tdTomato* |
| SL239' | *h+* | *leu1- ade6-M216 cen2::lacO-kanR:ura+ his7+::P.dis1-GFP-lacI-NLS cen3::Scer\LEU2:tetO* |
|  |  | *zfs1+::natR:P.adh31-tetR-tdTomato* |
| SL231 | *h+* | *leu1- ade6-M216 cen2::lacO-kanR:ura+ his7+::P.dis1-GFP-lacI-NLS dh1L::ura4+:tetO zfs1+::natR:P.adh31-tetR-tdTomato cut9-665* |
| SX444 | *h?* | *leu1- ura4-D18? cen2::lacO-kanR:ura+ his7+::P.dis1-GFP-lacI-NLS dh1L::ura4+:tetO zfs1+::natR:P.adh31-tetR-tdTomato cut9-665* |
| SX445' | *h?* | *leu1- ade6-M216 ura4-D18? cen2::lacO-kanR:ura+ his7+::P.dis1-GFP-lacI-NLS dh1L::ura4+:tetO zfs1+::natR:P.adh31-tetR-tdTomato cut9-665* |
| SM386/SM386'/ SM386'' | *h?* | *leu1- ade6-M216 cen2::lacO-kanR:ura+ his7+::P.dis1-GFP-lacI-NLS cen3::Scer\LEU2:tetO zfs1+::natR:P.adh31-tetR-tdTomato cut9-665* |
| **Figure 4D** |  |  |
| SI541 | *h+* | *leu1- ade6-M216 ura4-D18? cen2::lacO-kanR:ura+ his7+::P.dis1-GFP-lacI-NLS dh1L::ura4+:tetO zfs1+::natR:P.adh31-tetR-tdTomato* |
| SL239' | *h+* | *leu1- ade6-M216 cen2::lacO-kanR:ura+ his7+::P.dis1-GFP-lacI-NLS cen3::Scer\LEU2:tetO zfs1+::natR:P.adh31-tetR-tdTomato* |
| **Figure 5B** |  |  |
| SI541 | *h+* | *leu1- ade6-M216 ura4-D18? cen2::lacO-kanR:ura+ his7+::P.dis1-GFP-lacI-NLS dh1L::ura4+:tetO zfs1+::natR:P.adh31-tetR-tdTomato* |
| SL239' | *h+* | *leu1- ade6-M216 cen2::lacO-kanR:ura+ his7+::P.dis1-GFP-lacI-NLS cen3::Scer\LEU2:tetO zfs1+::natR:P.adh31-tetR-tdTomato* |
| **Figure S1A-D** |  |  |
| SL249 | *h-* | *leu1- ade6-M216 dh1L::ura4+:tetO zfs1+::natR:P.adh31-tetR-tdTomato cut2-GFP:kanR* |
| **Figure S1E** |  |  |
| SI541 | *h+* | *leu1- ade6-M216 ura4-D18? cen2::lacO-kanR:ura+ his7+::P.dis1-GFP-lacI-NLS dh1L::ura4+:tetO zfs1+::natR:P.adh31-tetR-tdTomato* |
| **Figure S1F** |  |  |
| SU495 | *h-* | *leu1- ura4-D18? FY397::RS imr1L::lacO-RS his7+::P.dis1-GFP-lacI-NLS dh1R::tetO:ura4+ zfs1+::natR:P.adh31-tetR-tdTomato* |
| **Figure S1G** |  |  |
| SI541 | *h+* | *leu1- ade6-M216 ura4-D18? cen2::lacO-kanR:ura+ his7+::P.dis1-GFP-lacI-NLS dh1L::ura4+:tetO zfs1+::natR:P.adh31-tetR-tdTomato* |
| SL239' | *h+* | *leu1- ade6-M216 cen2::lacO-kanR:ura+ his7+::P.dis1-GFP-lacI-NLS cen3::Scer\LEU2:tetO zfs1+::natR:P.adh31-tetR-tdTomato* |

| **Figure S1H** |  |  |
| --- | --- | --- |
| SI541 | *h+* | *leu1- ade6-M216 ura4-D18? cen2::lacO-kanR:ura+ his7+::P.dis1-GFP-lacI-NLS dh1L::ura4+:tetO zfs1+::natR:P.adh31-tetR-tdTomato* |
| ST653'' | *h+* | *leu1- ura4-D18? dh1L::lacO:ura4+ his7+::P.dis1-GFP-lacI-NLS cen2::Scer\LEU2:tetO zfs1+::natR:P.adh31-tetR-tdTomato* |
| **Figure S1I,K** |  |  |
| SU287 | *h90* | *leu1- ura4-D18? ade8∆::kanR:ura4:lacO his7+::P.dis1-GFP-lacI-NLS cnt2::tetO:ura4+ zfs1+::natR:P.adh31-tetR-tdTomato* |
| **Figure S1J** |  |  |
| SU286 | *h90* | *leu1- ura4-D18? ade8∆::kanR:ura4:lacO his7+::P.dis1-GFP-lacI-NLS cen2::Scer\LEU2:tetO zfs1+::natR:P.adh31-tetR-tdTomato* |
| **Figure S2A-B,E** |  |  |
| SI541 | *h+* | *leu1- ade6-M216 ura4-D18? cen2::lacO-kanR:ura+ his7+::P.dis1-GFP-lacI-NLS dh1L::ura4+:tetO zfs1+::natR:P.adh31-tetR-tdTomato* |
| SL239' | *h+* | *leu1- ade6-M216 cen2::lacO-kanR:ura+ his7+::P.dis1-GFP-lacI-NLS cen3::Scer\LEU2:tetO zfs1+::natR:P.adh31-tetR-tdTomato* |
| SM388 | *h?* | *leu1- ade6-M21? ura4-D18? cen2::lacO-kanR:ura+ his7+::P.dis1-GFP-lacI-NLS dh1L::ura4+:tetO zfs1+::natR:P.adh31-tetR-tdTomato cut1-206* |
| SM387 | *h?* | *leu1- ade6-M21? cen2::lacO-kanR:ura+ his7+::P.dis1-GFP-lacI-NLS cen3::Scer\LEU2:tetO zfs1+::natR:P.adh31-tetR-tdTomato cut1-206* |
| **Figure S2C-D** |  |  |
| SM388 | *h?* | *leu1- ade6-M21? ura4-D18? cen2::lacO-kanR:ura+ his7+::P.dis1-GFP-lacI-NLS dh1L::ura4+:tetO zfs1+::natR:P.adh31-tetR-tdTomato cut1-206* |
| **Figure S3A-B** |  |  |
| SL249 | *h-* | *leu1- ade6-M216 dh1L::ura4+:tetO zfs1+::natR:P.adh31-tetR-tdTomato cut2-GFP:kanR* |
| SM325' | *h-* | *leu1- ade6-M216 ura4-D18? dh1L::ura4+:tetO zfs1+::natR:P.adh31-tetR-tdTomato natNT2:P.adh1(#6)-cut2-GFP:kanR* |
| SW503 | *h-* | *leu1- ade6-M216 ura4-D18? dh1L::ura4+:tetO zfs1+::natR:P.adh31-tetR-tdTomato natNT2:P.adh1(#6)-cut2-GFP:kanR hphNT1:P.ark1-cut1+* |
| SW502 | *h-* | *leu1+::P.cut1(long)-cut1+ ade6-M216 ura4-D18? dh1L::ura4+:tetO zfs1+::natR:P.adh31-tetR-tdTomato natNT2:P.adh1(#6)-cut2-GFP:kanR* |
| **Figure S3C,E** |  |  |
| SI541 | *h+* | *leu1- ade6-M216 ura4-D18? cen2::lacO-kanR:ura+ his7+::P.dis1-GFP-lacI-NLS dh1L::ura4+:tetO zfs1+::natR:P.adh31-tetR-tdTomato* |
| SL239' | *h+* | *leu1- ade6-M216 cen2::lacO-kanR:ura+ his7+::P.dis1-GFP-lacI-NLS cen3::Scer\LEU2:tetO zfs1+::natR:P.adh31-tetR-tdTomato* |
| SW560 | *h-* | *leu1- cen2::lacO-kanR:ura+ his7+::P.dis1-GFP-lacI-NLS dh1L::ura4+:tetO zfs1+::natR:P.adh31-tetR-tdTomato natNT2:P.adh1(#6)-cut2-GFP(Y66L):kanR* |
| SW559 | *h+* | *leu1- cen2::lacO-kanR:ura+ his7+::P.dis1-GFP-lacI-NLS cen3::Scer\LEU2:tetO zfs1+::natR:P.adh31-tetR-tdTomato natNT2:P.adh1(#6)-cut2-GFP(Y66L):kanR* |
| **Figure S3D,F** |  |  |
| SW560 | *h-* | *leu1- cen2::lacO-kanR:ura+ his7+::P.dis1-GFP-lacI-NLS dh1L::ura4+:tetO zfs1+::natR:P.adh31-tetR-tdTomato natNT2:P.adh1(#6)-cut2-GFP(Y66L):kanR* |
| SW559 | *h+* | *leu1- cen2::lacO-kanR:ura+ his7+::P.dis1-GFP-lacI-NLS cen3::Scer\LEU2:tetO zfs1+::natR:P.adh31-tetR-tdTomato natNT2:P.adh1(#6)-cut2-GFP(Y66L):kanR* |
| SW562 | *h-* | *leu1+::P.cut1(long)-cut1+ cen2::lacO-kanR:ura+ his7+::P.dis1-GFP-lacI-NLS dh1L::ura4+:tetO zfs1+::natR:P.adh31-tetR-tdTomato natNT2:P.adh1(#6)-cut2-GFP(Y66L):kanR* |
| SW566 | *h-* | *leu1+::P.cut1(long)-cut1+ cen2::lacO-kanR:ura+ his7+::P.dis1-GFP-lacI-NLS cen3::Scer\LEU2:tetO zfs1+::natR:P.adh31-tetR-tdTomato natNT2:P.adh1(#6)-cut2-GFP(Y66L):kanR* |
| **Figure S4A** |  |  |
| SW596 | *h-* | *cen3::Scer\LEU2:tetO zfs1+::natR:P.adh31-tetR-tdTomato leu1-32::SV40-GFP-atb2[LEU1]* |

| **Figure S4B** |  |  |
| --- | --- | --- |

| SI541 | *h+* | *leu1- ade6-M216 ura4-D18? cen2::lacO-kanR:ura+ his7+::P.dis1-GFP-lacI-NLS dh1L::ura4+:tetO* |
| --- | --- | --- |
|  |  | *zfs1+::natR:P.adh31-tetR-tdTomato* |
| SL239' | *h+* | *leu1- ade6-M216 cen2::lacO-kanR:ura+ his7+::P.dis1-GFP-lacI-NLS cen3::Scer\LEU2:tetO zfs1+::natR:P.adh31-tetR-tdTomato* |
| **Figure S4C** |  |  |
| SI541 | *h+* | *leu1- ade6-M216 ura4-D18? cen2::lacO-kanR:ura+ his7+::P.dis1-GFP-lacI-NLS dh1L::ura4+:tetO zfs1+::natR:P.adh31-tetR-tdTomato* |
| SL239' | *h+* | *leu1- ade6-M216 cen2::lacO-kanR:ura+ his7+::P.dis1-GFP-lacI-NLS cen3::Scer\LEU2:tetO zfs1+::natR:P.adh31-tetR-tdTomato* |
| SX614 | *h+* | *leu1- ade6-M216 ura4-D18? cen2::lacO-kanR:ura+ his7+::P.dis1-GFP-lacI-NLS dh1L::ura4+:tetO zfs1+::natR:P.adh31-tetR-tdTomato klp5Δ::hygR* |
| SX615 | *h+* | *leu1- ade6-M216 cen2::lacO-kanR:ura+ his7+::P.dis1-GFP-lacI-NLS cen3::Scer\LEU2:tetO zfs1+::natR:P.adh31-tetR-tdTomato klp5Δ::hygR* |
| **Figure S4D** |  |  |
| SX614 | *h+* | *leu1- ade6-M216 ura4-D18? cen2::lacO-kanR:ura+ his7+::P.dis1-GFP-lacI-NLS dh1L::ura4+:tetO zfs1+::natR:P.adh31-tetR-tdTomato klp5Δ::hygR* |
| **Figure S4E** |  |  |
| SL249 | *h-* | *leu1- ade6-M216 dh1L::ura4+:tetO zfs1+::natR:P.adh31-tetR-tdTomato cut2-GFP:kanR* |
| SL253 | *h-* | *leu1+::P.nmt81-cdc13Δ(1-67) ade6-M216 dh1L::ura4+:tetO zfs1+::natR:P.adh31-tetR-tdTomato cut2-GFP* |
| **Figure S4F** |  |  |
| SL249 | *h-* | *leu1- ade6-M216 dh1L::ura4+:tetO zfs1+::natR:P.adh31-tetR-tdTomato cut2-GFP:kanR* |
| SL275 | *h-* | *leu1+::P.nmt81-cut2Δ(1-75) ade6-M216 dh1L::ura4+:tetO zfs1+::natR:P.adh31-tetR-tdTomato cut2-GFP:kanR* |
| **Figure S4H-J** |  |  |
| SL249 | *h-* | *leu1- ade6-M216 dh1L::ura4+:tetO zfs1+::natR:P.adh31-tetR-tdTomato cut2-GFP:kanR* |
| SL258' | *h-* | *leu1- ade6-M216 dh1L::ura4+:tetO zfs1+::natR:P.adh31-tetR-tdTomato cut9-665 cut2-GFP:kanR* |
| **Figure S4K** |  |  |
| ST968 | *h+* | *leu1- ura4-D18 cnt3::Scer\LEU2:lacO his7+::P.dis1-GFP-lacI-NLS cnt2::tetO:ura4+ zfs1+::natR:P.adh31-tetR-tdTomato* |
| **Figure S5A** |  |  |
| SI541 | *h+* | *leu1- ade6-M216 ura4-D18? cen2::lacO-kanR:ura+ his7+::P.dis1-GFP-lacI-NLS dh1L::ura4+:tetO* |
|  |  | *zfs1+::natR:P.adh31-tetR-tdTomato* |
| SL239' | *h+* | *leu1- ade6-M216 cen2::lacO-kanR:ura+ his7+::P.dis1-GFP-lacI-NLS cen3::Scer\LEU2:tetO zfs1+::natR:P.adh31-tetR-tdTomato* |
| SM388 | *h?* | *leu1- ade6-M21? ura4-D18? cen2::lacO-kanR:ura+ his7+::P.dis1-GFP-lacI-NLS dh1L::ura4+:tetO zfs1+::natR:P.adh31-tetR-tdTomato cut1-206* |
| SM387 | *h?* | *leu1- ade6-M21? cen2::lacO-kanR:ura+ his7+::P.dis1-GFP-lacI-NLS cen3::Scer\LEU2:tetO zfs1+::natR:P.adh31-tetR-tdTomato cut1-206* |
| SL231 | *h+* | *leu1- ade6-M216 cen2::lacO-kanR:ura+ his7+::P.dis1-GFP-lacI-NLS dh1L::ura4+:tetO zfs1+::natR:P.adh31-tetR-tdTomato cut9-665* |
| SX444 | *h?* | *leu1- ura4-D18? cen2::lacO-kanR:ura+ his7+::P.dis1-GFP-lacI-NLS dh1L::ura4+:tetO zfs1+::natR:P.adh31-tetR-tdTomato cut9-665* |
| SX445' | *h?* | *leu1- ade6-M216 ura4-D18? cen2::lacO-kanR:ura+ his7+::P.dis1-GFP-lacI-NLS dh1L::ura4+:tetO zfs1+::natR:P.adh31-tetR-tdTomato cut9-665* |
| SM386/SM386'/ SM386'' | *h?* | *leu1- ade6-M216 cen2::lacO-kanR:ura+ his7+::P.dis1-GFP-lacI-NLS cen3::Scer\LEU2:tetO zfs1+::natR:P.adh31-tetR-tdTomato cut9-665* |
