# Supplementary material for: Chromosome segregation synchrony in S. pombe is noise limited and arises without positive feedback
Source: J Cell Biol. 2026 May 14;225(7):e202602088. doi: 10.1083/jcb.202602088 (PMC13175033; doi:10.1083/jcb.202602088)
Supplement: Table S3 — shows stochastic model variants. [file jcb_202602088_tables3.docx]

Table S3 – Stochastic model variants

|  | **Features** | **Parameters** | **Separase activity ramp** |
| --- | --- | --- | --- |
| **Basic model** |  | *N*_2_, *n*_2_, *R*_12_, *R*_32_, *r*_12_, *r*_32_, *k*_max_, τ  Perturbation conditions:  α, β_k_, β_τ_, β_τ2_ | slow, 2 < τ < 240 |
|  |  |  | fast, 0.5 < τ < 5 |
| **Processive separase**  **action** | *b* cohesin molecules removed within one  removal event | same as basic model, and *b* | slow, 2 < τ < 240 |
|  |  |  | fast, 0.5 < τ < 5 |
| **Steric Hindrance** | effective *k* scales with surface-to-volume ratio  (Eq. 6) | same as basic model, and *n*_inner_ | slow, 2 < τ < 240 |
|  |  |  | fast, 0.5 < τ < 5 |
