# Supplementary material for: Chromosome segregation synchrony in S. pombe is noise limited and arises without positive feedback
Source: J Cell Biol. 2026 May 14;225(7):e202602088. doi: 10.1083/jcb.202602088 (PMC13175033; doi:10.1083/jcb.202602088)
Supplement: Table S4 — shows model parameters and their bounds for fitting. [file jcb_202602088_tables4.docx]

# Table S4 - Model parameters and their bounds for fitting

| **Symbol** | **Description** | **Unit** | **Parameter Bounds** | **Parameter Justification** |
| --- | --- | --- | --- | --- |
| *N*_2_ | Initial cohesin count for the reference chromosome (chromosome 2) | 1 | [50 - 1000] | Range was set broadly to include (i) cohesin-binding patterns in fission yeast (Schmidt et al., 2009; Mizuguchi et al., 2014), (ii) absolute protein abundance showing cohesin subunits exist at 10^2^-10^3^ or more molecules per cell (Marguerat et al., 2012; Carpy et al., 2014), and (iii) additional quantification showing large numbers of cohesin complex complexes in other cell types, both cohesive and non-cohesive (Holzmann et al., 2019), and (iv) the fact that not all cohesin contributes to cohesion (Gerlich et al., 2006; Feytout et al., 2011; Tomonaga et al., 2000). |
| *n*_2_ | Cohesin threshold count for the reference chromosome (chromosome 2) | 1 | [0 - 50] | Low number regime reflects that cohesion may persist with few cohesin complexes, consistent with evidence that (i) only a small pool of cohesive cohesin needs to be removed at anaphase (Tomonaga et al., 2000), (ii) chromosome separation is normal with substantially reduced cohesin levels (Heidinger-Pauli et al., 2010), and (iii) that chromosomes fail to separate with substantial cohesin but begin to separate with lower levels of cohesin (Carvalhal et al., 2018).  Upper bound allows thresholds that reflect (i) estimates of spindle pulling forces (Grishchuk et al., 2005; Chacón et al., 2014; Gudimchuk and Alexandrova, 2023; Akiyoshi et al., 2010), (ii) the number of microtubules attached to fission yeast kinetochores (Ding et al., 1993), (iii) the force required to mechanically break cohesin (Richeldi et al., 2024), and (iv) potential impacts of cohesion fatigue in response to spindle forces  (Sapkota et al., 2018; Daum et al., 2011). |
| *R*_12_ | Ratio for the initial cohesin counts of chromosome 1 over chromosome 2 (*N*_1_/*N*_2_) | 1 | [0.4 - 2] | Allows chromosome I and chromosome III to have 0.4 to 2x or 0.5 to 5x the amount of starting cohesin relative to chromosome II, respectively, based on (i) genome-wide cohesin binding patterns in fission yeast (Schmidt et al., 2009; Mizuguchi et al., 2014),  (ii) the fact that centromere organization may influence cohesin levels (Paldi et al., 2020; Yeh et  al., 2008), and (iii) assumptions that the size of centromeres may influence cohesin load (Nonaka et al., 2002; Bernard et al., 2001). |
| *R*_32_ | Ratio for the initial cohesin counts of chromosome 3 over chromosome 2 (*N*_3_/*N*_2_) | 1 | [0.5 - 5] |  |
| *r*_12_ | Ratio for the cohesin threshold of chromosome 1 over chromosome 2 (*n*_1_/*n*_2_) | 1 | [0.25 - 4] | Range is set to allow an up to 4-fold difference in effective threshold between chromosomes, based on the observed 2-4 microtubules per kinetochore in fission yeast (Ding et al., 1993; Joglekar et al., 2008). |
| *r*_32_ | Ratio for the cohesin threshold of chromosome 3 over chromosome 2 (*n*_3_/*n*_2_) | 1 | [0.25 - 4] |  |
| *k*_max_ | Maximum cohesin degradation rate | sec^-1^ | [0.001 - 0.1] | Range spans a large scale of possible maximum rates of cohesin cleavage. |
| τ | Time to reach *k*_max_ | sec | [2 - 240]  [0.5 - 5] for separase autoactivation | Range spans a minutes- to seconds-scale rate of separase activation, to allow gradual activity ramps or rapid separase activation.  To mimic separase autoactivation, the range is constrained to seconds scale. |
| *b* | Number of cohesin molecules removed per event | 1 | [1 - 50] | Processive separase action model-specific parameter. Range allows small or large “bursts” of separase activity. |
| *n*_inner_ | Number of cohesin molecules in the innermost core, the region without steric hindrance | 1 | [1 - 100] | Steric hindrance model-specific parameter.  Range allows a subset of cohesin in the innermost core, where it is not shielded by steric hindrance,  but remains well below the upper bound of *N*_2_. |
| α | Multiplier modifying the  cohesin threshold (*n*_2_) | 1 | [0.1 - 0.7] | Constrains MBC treatment conditions to lower *n*_2_  range to reflect reduced microtubule forces. |
| β_k_ | Multiplier modifying *k*_max_ | 1 | [0.1 - 1] | Allows lower *k*_max_ in separase mutant conditions to reflect reduced separase activity. |
| β_τ_ | Multiplier modifying the activation timescale (τ) to mimic APC/C mutants | 1 | [1 - 10]  [1 - 3] for separase autoactivation | Allows higher τ in APC/C mutant and velcade treatment conditions to reflect slower separase activation. |
| β_τ2_ | Multiplier modifying the activation timescale (τ) to mimic velcade treatment | 1 | [1 - 20]  [1 - 3] for separase autoactivation |  |

References:

Akiyoshi, B., K.K. Sarangapani, A.F. Powers, C.R. Nelson, S.L. Reichow, H. Arellano-Santoyo, T. Gonen, J.A. Ranish, C.L. Asbury, and S. Biggins. 2010. Tension directly stabilizes reconstituted kinetochore-microtubule attachments. Nature. 468:576–579. doi:10.1038/nature09594.

Bernard, P., J.-F. Maure, J.F. Partridge, S. Genier, J.-P. Javerzat, and R.C. Allshire. 2001. Requirement of Heterochromatin for Cohesion at Centromeres. Science. 294:2539–2542. doi:10.1126/science.1064027.

Carpy, A., K. Krug, S. Graf, A. Koch, S. Popic, S. Hauf, and B. Macek. 2014. Absolute Proteome and Phosphoproteome Dynamics during the Cell Cycle of Schizosaccharomyces pombe (Fission Yeast)*. Mol. Cell. Proteom. 13:1925–1936. doi:10.1074/mcp.m113.035824.

Carvalhal, S., A. Tavares, M.B. Santos, M. Mirkovic, and R.A. Oliveira. 2018. A quantitative analysis of cohesin decay in mitotic fidelity. J. Cell Biol. 217:3343–3353. doi:10.1083/jcb.201801111.

Chacón, J.M., S. Mukherjee, B.M. Schuster, D.J. Clarke, and M.K. Gardner. 2014. Pericentromere tension is self-regulated by spindle structure in metaphase. J. Cell Biol. 205:313–324. doi:10.1083/jcb.201312024.

Daum, J.R., T.A. Potapova, S. Sivakumar, J.J. Daniel, J.N. Flynn, S. Rankin, and G.J. Gorbsky. 2011. Cohesion Fatigue Induces Chromatid Separation in Cells Delayed at Metaphase. Curr. Biol. 21:1018–1024. doi:10.1016/j.cub.2011.05.032.

Ding, R., K.L. McDonald, and J.R. McIntosh. 1993. Three-dimensional reconstruction and analysis of mitotic spindles from the yeast, Schizosaccharomyces pombe. J. Cell Biol. 120:141–151. doi:10.1083/jcb.120.1.141.

Feytout, A., S. Vaur, S. Genier, S. Vazquez, and J.-P. Javerzat. 2011. Psm3 Acetylation on Conserved Lysine Residues Is Dispensable for Viability in Fission Yeast but Contributes to Eso1-Mediated Sister Chromatid Cohesion by Antagonizing Wpl1. Mol Cell Biol. 31:1771–1786. doi:10.1128/mcb.01284-10.

Gerlich, D., B. Koch, F. Dupeux, J.-M. Peters, and J. Ellenberg. 2006. Live-Cell Imaging Reveals a Stable Cohesin-Chromatin Interaction after but Not before DNA Replication. Curr. Biol. 16:1571–1578. doi:10.1016/j.cub.2006.06.068.

Grishchuk, E.L., M.I. Molodtsov, F.I. Ataullakhanov, and J.R. McIntosh. 2005. Force production by disassembling microtubules. Nature. 438:384–388. doi:10.1038/nature04132.

Gudimchuk, N.B., and V.V. Alexandrova. 2023. Measuring and modeling forces generated by microtubules.

Biophys. Rev. 15:1095–1110. doi:10.1007/s12551-023-01161-7.

Heidinger-Pauli, J.M., O. Mert, C. Davenport, V. Guacci, and D. Koshland. 2010. Systematic Reduction of Cohesin Differentially Affects Chromosome Segregation, Condensation, and DNA Repair. Curr. Biol. 20:957–963. doi:10.1016/j.cub.2010.04.018.

Holzmann, J., A.Z. Politi, K. Nagasaka, M. Hantsche-Grininger, N. Walther, B. Koch, J. Fuchs, G. Dürnberger, W. Tang, R. Ladurner, R.R. Stocsits, G.A. Busslinger, B. Novák, K. Mechtler, I.F. Davidson, J. Ellenberg, and J.-M. Peters. 2019. Absolute quantification of cohesin, CTCF and their regulators in human cells. eLife. 8:e46269. doi:10.7554/elife.46269.

Joglekar, A.P., D. Bouck, K. Finley, X. Liu, Y. Wan, J. Berman, X. He, E.D. Salmon, and K.S. Bloom. 2008.

Molecular architecture of the kinetochore-microtubule attachment site is conserved between point and regional centromeres. J. Cell Biol. 181:587–594. doi:10.1083/jcb.200803027.

Marguerat, S., A. Schmidt, S. Codlin, W. Chen, R. Aebersold, and J. Bähler. 2012. Quantitative Analysis of Fission Yeast Transcriptomes and Proteomes in Proliferating and Quiescent Cells. Cell. 151:671–683. doi:10.1016/j.cell.2012.09.019.

Mizuguchi, T., G. Fudenberg, S. Mehta, J.-M. Belton, N. Taneja, H.D. Folco, P. FitzGerald, J. Dekker, L. Mirny, J. Barrowman, and S.I.S. Grewal. 2014. Cohesin-dependent globules and heterochromatin shape 3D genome architecture in S. pombe. Nature. 516:432–435. doi:10.1038/nature13833.

Nonaka, N., T. Kitajima, S. Yokobayashi, G. Xiao, M. Yamamoto, S.I.S. Grewal, and Y. Watanabe. 2002. Recruitment of cohesin to heterochromatic regions by Swi6/HP1 in fission yeast. Nat Cell Biol. 4:89–93. doi:10.1038/ncb739.

Paldi, F., B. Alver, D. Robertson, S.A. Schalbetter, A. Kerr, D.A. Kelly, J. Baxter, M.J. Neale, and A.L. Marston. 2020. Convergent genes shape budding yeast pericentromeres. Nature. 582:119–123. doi:10.1038/s41586-020-2244-6.

Richeldi, M., G. Pobegalov, T.L. Higashi, K. Gmurczyk, F. Uhlmann, and M.I. Molodtsov. 2024. Mechanical disengagement of the cohesin ring. Nat. Struct. Mol. Biol. 31:23–31. doi:10.1038/s41594-023-01122-4.

Sapkota, H., E. Wasiak, J.R. Daum, and G.J. Gorbsky. 2018. Multiple determinants and consequences of cohesion fatigue in mammalian cells. Mol. Biol. Cell. 29:1811–1824. doi:10.1091/mbc.e18-05-0315.

Schmidt, C.K., N. Brookes, and F. Uhlmann. 2009. Conserved features of cohesin binding along fission yeast chromosomes. Genome Biol. 10:R52. doi:10.1186/gb-2009-10-5-r52.

Tomonaga, T., K. Nagao, Y. Kawasaki, K. Furuya, A. Murakami, J. Morishita, T. Yuasa, T. Sutani, S.E. Kearsey, F. Uhlmann, K. Nasmyth, and M. Yanagida. 2000. Characterization of fission yeast cohesin: essential anaphase proteolysis of Rad21 phosphorylated in the S phase. Genes Dev. 14:2757–2770. doi:10.1101/gad.832000.

Yeh, E., J. Haase, L.V. Paliulis, A. Joglekar, L. Bond, D. Bouck, E.D. Salmon, and K.S. Bloom. 2008. Pericentric Chromatin Is Organized into an Intramolecular Loop in Mitosis. Curr Biol. 18:81–90. doi:10.1016/j.cub.2007.12.019.
